# Is it still worthwhile to perform quarterly cd4+ t lymphocyte cell counts on hiv-1 infected stable patients?

**DOI:** 10.1186/s12879-017-2199-x

**Published:** 2017-02-06

**Authors:** Antonio Di Biagio, Marta Ameri, Davide Sirello, Giovanni Cenderello, Enrico Di Bella, Lucia Taramasso, Barbara Giannini, Mauro Giacomini, Claudio Viscoli, Giovanni Cassola, Marcello Montefiori

**Affiliations:** 1 Infectious Disease Clinic, IRCCS San Martino – IST Hospital, Genoa, Italy; 2 Department of Economics, University of Genoa, Via Vivaldi 5, 16126 Genoa, Italy; 3 Infectious Diseases Unit, EO Galliera, Genoa, Italy; 4 Department of Informatics, Bioengineering, Robotics and System Engineering, University of Genoa, Genoa, Italy

**Keywords:** Lymphocyte T CD4+, Cost, HIV, Monitoring

## Abstract

**Background:**

In the last 20 years routine T CD4+ lymphocyte (CD4+) cell count has proved to be a key factor to determine the stage of HIV infection and start or discontinue of prophylaxis for opportunistic infections. However, several studies recently showed that in stable patients on cART a quarterly CD4+ cell count monitoring results in limited (or null) clinical relevance. The research is intended to investigate whether performing quarterly CD4+ cell counts in stable HIV-1 patients is still recommendable and to provide a forecast of the cost saving that could be achieved by reducing CD4+ monitoring in such a category of patients.

**Methods:**

The study is based on data referring to all HIV-infected patients > 18 years of age being treated at two large infectious diseases units located in the metropolitan area of Genoa, Italy. The probability of CD4+ cell counts dropping below a threshold value set at 350 cells/mm^3^ is assessed using confidence intervals and Kaplan-Meier survival estimates, whereas multivariate Cox analysis and logistic regression are implemented in order to identify factors associated with CD4+ cell count falls below 350 cells/mm^3^.

**Results:**

Statistical analysis reveals that among stable patients the probability of maintaining CD4+ >350 cell/mm^3^ is more than 98%. Econometric models indicate that HCV co-infection and HIV-RNA values >50 copies/mL in previous examinations are associated with CD4+ falls below 350 cells/mm^3^. Moreover, results suggest that the cost saving that could be obtained by reducing CD4+ examinations ranges from 33 to 67%.

**Conclusions:**

Empirical findings shows that patients defined as stable at enrollment are highly unlikely to experience a CD4+ value <350 cell/mm^3^ in the space/arc of a year. The research supports a recommendation for annual CD4+ monitoring in stable HIV-1 patients.

## Background

CD4+ T lymphocyte (CD4+) cell count monitoring is playing a crucial role as a surrogate marker of the immune system function in the clinical management of Human Immunodeficiency Virus (HIV) infection [1–3]. In the last 20 years, routine CD4+ cell counts, in combination with HIV-RNA copies counts [4], have proved to be the best predictors of therapy effectiveness and probability of disease progression [5–7]. Consistently with these findings, both the World Health Organization (WHO) and the United States Department of Health and Human Services (DHHS) recommend HIV-RNA as the most valuable marker of combined antiretroviral therapy (cART) efficacy [8, 9]. Indeed, CD4+ cell count is still a key factor in determining the stage of HIV infection [9] and plays a role in guiding clinical care to start or discontinue the prophylaxis for opportunistic infections [10]. It is widely accepted by the scientific community that the risk of onset of opportunistic infection increases considerably as the CD4+ falls below 350 cells/mm^3^ [11], even if the primary prophylaxis starts at 200 cells/mm^3^ [12–14]. However, in patients on cART with CD4+ >200 cell/mm^3^ and HIV-RNA <50 copies/mL, several studies have shown that quarterly CD4+ cell count monitoring results in limited (or null) clinical relevance [15–23] and so a less frequent (e.g. yearly) CD4+ monitoring strategy has been proposed for this patient category. A reduction in the frequency of CD4+ cell counts could lead to considerable financial savings. A recent study [24], estimated that the adoption of a once per year CD4+ monitoring strategy for all patients with HIV-RNA <50 copies/mL in the United States could result in annual savings of 10.2 million United States dollars (USD). A similar study, carried out in Australia outlined potential financial savings of 1.4 million (USD) by adopting the once a year CD4+ monitoring strategy for all the eligible Australian patients with HIV infection [21].

In this paper we report the results of a retrospective observational study in a cohort of HIV-infected patients with the objective to show how often, in virologically suppressed patients, a decrease CD4+ cell count was observed and, therefore, if performing quarterly CD4+ counts is still recommendable.

## Methods

This was a retrospective, longitudinal, multicenter analysis of 1771 HIV-infected patients followed in two large infectious diseases units located in the metropolitan area of Genoa (Liguria Region), Italy.

Patients are enrolled in the RETE LIGURE HIV database after giving informed consent to provide their data for academic no profit studies, (Ethical Committee Liguria Region, August 28, 2013). The data include demographics (age, gender, nationality), hepatitis C virus (HCV) - co-infection (defined as HCV-antibody positivity) risk factors for HIV infection, time since HIV infection and both HIV-RNA and CD4+ cell count status. All data were collected through a web-based platform (www.reteligureHIV.it).

Eligible patients for the study were HIV-infected adults, with HIV-RNA <50 copies/mL and CD4+ >500 cell/mm^3^ throughout 2011. These patients, defined as “stable patients”, were included in the study and observed during a 1-year time period, from January to December 2012.

The primary endpoint was investigating whether there was any significant change in CD4+ cell counts in stable patients. Since HIV-RNA <50 copies/mL leads to rises in CD4+ cell counts and improved survival, particularly if the CD4+ cell count has risen above 350 cells/mm^3^ [25], the value of 350 cells/mm3 was set as the threshold [26].

According to previous studies [16], confidence intervals and Kaplan-Meier survival estimates were implemented and the probability of the CD4+ cell count dropping below the threshold value was assessed. Moreover, a multivariate Cox analysis and a logistic regression were used in order to identify factors associated with CD4+ cell count falls below the threshold value of 350 cells/mm^3^.

A forecast of the savings that could be achieved by reducing CD4+ monitoring in stable patients was finally provided. The economic consequences of less frequent CD4+ cell counts were estimated assuming two alternative scenarios: in the first one it was assumed that all stable patients were monitored once annually, while in the second model stable patients were divided on the basis of the results of the econometric analysis and it was hypothesized CD4+ cell counts twice a year for those patients characterized by higher risk of CD4+ declines below 350 cells/mm^3^. Referring to the national context, the total number of patients on cART was estimated according to Raimondo et al. [27].

The cost of each examination was computed using the official tariff established by the Italian Ministry of Health for CD4+ tests (€ 17.09 per test).

## Results

A total of 372 patients (21% of the entire cohort) met the inclusion criteria for study entry. The mean age was 51 years old, 61% of patients were male and 88% were Italians; the majority (73%) had been infected with HIV for more than 10 years, and 34% were HCV antibody positive (the demographic characteristics of the study population are shown in Table 1). During 2012 the mean number of CD4+ cell counts per patient was 2.7 with, on average, one examination every 107 days. During the period of observation, 7 out of 372 (1.88%) total stable HIV-infected patients showed CD4+ cell count values falling below 350 cells/mm^3^; on average, a CD4+ cell count value below 350 cells/mm^3^ was observed after 285 days (the first occurred after 51 days, while the last after 359 days). In details we registered two virological failures, three patients were treated with PEG-interferon and ribavirin, while two patients have a transient drop with complete recovery in the next control.

**Table 1:** Demographic and clinical characteristics of HIV-infected patients with HIV-RNA <50 copie/mL and CD4 > 500 cell/mm^3^

	Patients	%
Age		
< 45	72	19%
45–65	278	74%
65 +	26	7%
Gender		
Male	229	61%
Female	147	39%
Nationality		
Italian	329	88%
Other	47	12%
HCV co-infection		
Positive	126	34%
Negative	250	66%
Time since HIV infection (years)		
< 5	22	6%
5–10	79	21%
> 10	275	73%
Risk Factor		
Heterosexual	152	40%
IDU (a)	121	32%
MSM (b)	61	16%
Other	42	11%

(a) MSM men who have sex with men; (b) IDU injection drug users

Statistically significant differences in the frequency of CD4+ tests by age (<45 versus 45–65 and 65+, *p* = 0.01 and *p* = 0.003 respectively) and transmission risk (drug addiction versus other risk factors, *p* = 0.02) were detected by means of Kruskal-Wallis tests.

Table 2 provides the 95% binomial proportion confidence interval for the CD4+ count to fall below 350 cells/mm^3^: the probability of experiencing a CD4+ fall below 350 cells/mm^3^ ranges from 0.76 to 3.8%. When data were analyzed by HCV co-infection, it was noted that 1.2% (95%CI, 0.002–0.03) of non HCV co-infected patients had CD4+ values below 350 cells/mm^3^ in the arc of a year, while the percentage increased to 3.2% with reference to HCV co-infected patients (i.e. patients with HCV antibody positive) (95% CI, 0.01–0.08).

**Table 2:** 95% Confidence Interval of binomial variable CD4 < 350

Variable	Obs	Mean	Std. Err.	-- Binomial Exact --	
				[95% Conf. Interval]	
CD4 < 350	371	.0188679	.0070638	.0076187	.0384872

**Fig. 1:**
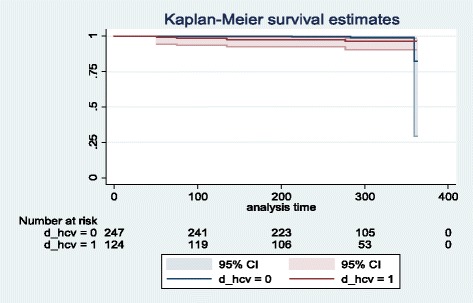
Kaplan-Meier survival estimates

As shown by the Kaplan-Meier survival estimates (Fig. 1), the probability of maintaining CD4+ ≥ 350 cells/mm^3^ was higher for patients without HCV co-infection (d_hcv = 0) rather than for those with co-infection (d_hcv = 1). Moreover, the data pointed out differences between the two groups of patients also with reference to the timing of CD4+ falls: on average, the CD4+ cell count value declines below 350 cells/mm^3^ in HCV co-infected patients occurred in 134 days, while in non-HCV in 285 days.

Results of the Cox regression (Table 3) suggested that CD4+ cell count value < 350 cells/mm^3^ was strongly defined (hazard ratio = 15.4) by HCV co-infection and HIV-RNA values > 50 copies/mL in the previous examination (hazard ratio = 5.95). The probability of having CD4+ < 350 cells/mm^3^ also seemed to be associated with being an injection drug user (hazard ratio = 0.03), a variable positively correlated with HCV co-infection (correlation index = 0.82), whereas the gender, age and time since HIV infection was detected did not seem to be determinant of CD4+ falling below 350 cells/mm^3^. These results were also confirmed by logistic regression (Table 3). HCV co-infection (Coef. = 2.2) and HIV-RNA values > 50 copies/mL in the previous examination (Coef. = 2.1) increased the probability of having CD4+ count value < 350 cells/mm^3^, while other variables did not impact significantly on CD4+ values.

**Table 3:** Cox regression and logit regression

Variable	Cox regression	Logit regression
	Hazard Ratio	Coefficient
Gender (1 = F)	.1764862 (0.143)	−1.728616 (0.159)
Age	.9883046 (0.749)	−.0174055 (0.649)
Dummy MSM (a)	.9057002 (0.934)	.0500618 (0.966)
Dummy IDU (b)	.035092 (0.005)***	−3.120108 (0.017)**
Dummy HCV co-infection	15.43625 (0.008)***	2.24666 (0.033)**
Time since HIV infection (years)	.9997115 (0.194)	−.0003251 (0.141)
HIV RNA load > 50 in previous tests	5.951844 (0.070)*	2.154811 (0.032)**
Constant	–	8.310305 (0.296)
	Log likehood = −28.6398	Log likelihood = −27.0727
	Prob. chi2 = 0.0336	Prob. chi2 = 0.0323

(a) MSM men who have sex with men; (b) IDU injection drug users

* *p* < 0.1

** *p* < 0.05

*** *p* < 0.01

In 2012 the total number of CD4+ cell counts among stable patients was 1002 and the global average of the CD4+ measurements was 2.7. The cost saving that could be obtained by reducing CD4+ examinations ranges from 33 to 67%.

The analysis demonstrated that if CD4+ monitoring of all stable patients was limited to once annually, 630 CD4+ measurements could be eliminated and the total annual expenditure for CD4+ examination could be reduced by 63% (1^st^ scenario). Furthermore, if CD4+ monitoring was limited to once annually in patients without HCV co-infection and twice annually in patients with co-infection, 506 CD4+ measurements could be cut and the total annual expenditure for CD4+ examinations could decrease by 50% (2^nd^ scenario). Moreover, compared with CD4+ frequency recommended by HIV antiretroviral Italian Guidelines (two or three times a year), the total cost saving could be 67% or 50% respectively in the first scenario, and 56% or 33% in the second one.

Table 4 reports the forecast of the impact of less frequent CD4+ monitoring of stable patients in the Italian context assuming the two alternative scenarios. The total cost saving is computed hypothesizing both the CD4+ frequency recommended by HIV antiretroviral Italian Guidelines (two or three times a year – column A and C) and the global average of CD4+ tests occurred in 2012 (column B). The results show that the reduction of CD4+ examinations could bring significant savings in economic resources, with values ranging from about € 200.000 to € 600.000.

**Table 4:** Economic consequences of less frequent CD4 monitoring in Italy

	Column A CD4 2 times/year	Column B CD4 2.7 times/year	Column C CD4 3 times/year
Total cost	€ 594.428	€ 799.506	€ 891.642
Cost saving (1st scenario)	€ 297.214	€ 502.292	€ 594.428
Cost saving (2nd scenario)	€ 198.143	€ 403.220	€ 495.357

## Discussion

The analysis suggests that yearly CD4+ monitoring in stable patients could be more appropriate.

During the period of observations none of the patients reached the critical value of 200 cells/mm^3^, which is usually considered as the threshold under which to start a prophylaxis against opportunistic infections (such as *Pneumocystis jirovecii* pneumonia) and no change in the therapies drawn by CD4+ cell count declines alone has been made in the study population. Furthermore, among the seven patients who fell below the threshold value, 6 rose again above 350 cells/mm^3^ in the next CD4+ cell count evaluation, and only one needed two rounds of tests before his CD4+ cell count was back above the threshold. Consequently, the hypothesis of a less frequent monitoring of CD4+ cell counts would not have had any consequence in terms of patient prognosis and therapies.

Indeed, an additional assurance for patient health is the preservation of HIV-RNA evaluation 3/4 times a year. An increase in HIV-RNA load may certainly represent a sort of “alarm bell” which strongly suggests a virological failure and, consequently, an immunological failure. HIV-RNA is the key factor in guiding therapy optimization: in patients with HIV-RNA <50 copies/mL HIV infection is unlikely to advance and the treatment does not require any change based on the one time reduction of CD4+ cell counts.

Our results probably underestimate the number of patients that could be monitored once a year: firstly, in our analysis we defined strict inclusion criteria for study entry and we set a threshold value at 350 cell/mm^3^, whereas previous research considered all patients with viral suppression and investigated the probability of CD4+ dropping below 200 cell/mm^3^; secondly, it is expected that in the near future the number of stable patients will increase, due to the new cART regimens, based on antiretroviral highly efficacy and better tolerated [28].

In this experience inclusion criteria were based only on immune-virological data and were independent from previous AIDS diagnosis and demographical or social variables. As a consequence, the results might be applicable to all patients who met the study criteria to define “stable”. However, a more frequent monitoring might be indicated also in these patients in case of changes in the clinical status or in case of concomitant treatments that could cause a CD4 + count reduction. Of note, the recent guidelines of Department of Health and Human Services [9], in patients who have been on cART since at 2 years, with HIV-RNA consistently suppressed, counsel to monitor CD4+ count yearly when CD4+ count is above 300 cells/mm^3^, or even not to monitor CD4+ count at all in patients with more than 500 CD4+ cells/mm^3^ (grade of recommendation CIII). According to our experience the crucial issues is the immunological “stability”, regardless the route of acquisition or the nadir cd4 count.

## Conclusions

The results of the statistical analysis reveal that among stable patients the probability of maintaining CD4+ >350 cell/mm^3^ was more than 98% and suggest that yearly CD4+ monitoring in this category of patients could be more appropriate.

Econometric models indicate that HCV co-infection and HIV-RNA load >50 copies/mL in previous examinations were associated with CD4+ falls below 350 cells/mm^3^. However, our data demonstrated that the risk of CD4+ cell count falling below 350 cell/mm^3^ was very low also in this scenario, as only 3.2% of the HCV co-infected patients had CD4+ values below 350 cells/mm^3^ in the space of a year.

Reduced CD4+ examinations would provide a significant cost saving: if all stable patients were monitored once per year, the total expenditure would be reduced by 63%, whereas it would be decreased by 50% by monitoring stable patients without HCV annually and those with HCV co-infection twice a year.

In conclusion, our results straighten the recommendation of reducing the frequency of CD4+ monitoring in stable patients highlighted by national and international guidelines, as this strategy revealed a potential cost-saving.

## Abbreviations

cART: Combined antiretroviral therapy
CD4+: CD4+ T Lymphocite
CI: Confidence interval
Coeff.: Coefficient
d_HCV: Dummy variable for HCV co-infection
HCV: Hepatitis C virus
HIV: Human Immunodeficiency Virus
Obs: Numbers of observations
Std. Err.: Standard error
USD: United States Dollars
